# Oral pre-exposure prophylaxis uptake, adherence, and adverse events among South African men who have sex with men and transgender women

**DOI:** 10.4102/sajhivmed.v23i1.1405

**Published:** 2022-11-08

**Authors:** Linda-Gail Bekker, Danielle Giovenco, Stefan Baral, Karen Dominguez, Rachel Valencia, Travis Sanchez, A.D. McNaghten, Ryan Zahn, Clarence S. Yah, Zinhle Sokhela, Richard Kaplan, Refliwe N. Phaswana-Mafuya, Chris Beyrer, Patrick S. Sullivan

**Affiliations:** 1 Desmond Tutu HIV Centre, Cape Town, South Africa; 2 Faculty of Health Sciences, University of Cape Town, Cape Town, South Africa; 3 International Health Institute, Brown University, Providence, United States of America; 4 Bloomberg School of Public Health, Johns Hopkins University, Baltimore, United States of America; 5 Contraceptive Research and Development (CONRAD), Eastern Virginia Medical School, Norfolk, United States of America; 6 Department of Epidemiology, Emory University, Atlanta, United States of America; 7 Department of Internal Medicine, Faculty of Health Sciences, University of the Witwatersrand, Johannesburg, South Africa; 8 Faculty of Health Sciences, School of Health System and Public Health, University of Pretoria, Pretoria, South Africa; 9 Wits Reproductive Health and HIV Institute, Faculty of Health Sciences, University of the Witwatersrand, Johannesburg, South Africa; 10 South African Medical Research Council/University of Johannesburg Pan African Centre for Epidemics Research, Faculty of Health Sciences, University of Johannesburg, Johannesburg, South Africa

**Keywords:** HIV, men who have sex with men, transgender women, sexually transmitted infections, pre-exposure prophylaxis, HIV prevention, South Africa

## Abstract

**Background:**

HIV prevention programmes that include pre-exposure prophylaxis (PrEP) for men who have sex with men (MSM) and transgender women (TGW) in South Africa have not been widely implemented.

**Objectives:**

The authors examined oral PrEP uptake, adherence, and adverse events among HIV-uninfected MSM and TGW to inform intervention acceptability and feasibility.

**Method:**

In 2015, MSM and TGW in two South African cities were offered a comprehensive package of HIV prevention services, including daily oral PrEP, and were followed for one year. Different models of PrEP delivery were used at each site. Adherence was measured using self-report and pill-count data and tenofovir-diphosphate (TFV-DP) concentrations.

**Results:**

Among 135 participants who were eligible for PrEP, 82 (61%) initiated PrEP, of whom 67 (82%) were on PrEP at study end. Participants were on PrEP for a median of 294 out of 314.5 possible days (93% protected days). The median time from PrEP initiation to discontinuation or study end was 305 days (interquartile range: 232–325 days). Across the follow-up time points, 57% – 72% of participants self-reported taking protective levels of PrEP and 59% – 74% were adherent to PrEP as indicated by pill counts. Fewer (≤ 18%) achieved protective TFV-DP concentrations of ≥ 700 fmol/punch in dried blood spots. Side effects, while typically mild, were the most commonly cited reason by participants for early PrEP discontinuation.

**Conclusion:**

Many MSM and TGW initiated and maintained PrEP, demonstrating that PrEP can be successfully delivered to South African MSM and TGW in diverse programmatic contexts. Biologic adherence measures suggest MSM and TGW may experience challenges taking PrEP regularly. Counselling for coping with side effects and motivating daily pill taking is recommended to support South African MSM and TGW in achieving protection with PrEP.

## Introduction

Globally, men who have sex with men (MSM) are disproportionately affected by HIV.^1,2,3^ In South Africa, the prevalence of HIV among MSM in South Africa is estimated to be 18.1%.^4^ This prevalence varies geographically, with estimates as high as 30% in Cape Town to 51% in Port Elizabeth.^5^ South African MSM face high levels of societal stigma and discrimination as a result of traditional and conservative attitudes within the general population.^6,7^ There is also a lack of knowledge and training around managing the particular health needs and vulnerabilities of MSM, making it especially difficult for MSM to obtain clinically and culturally competent sexual health and HIV prevention services.^8^ Comprehensive HIV prevention programmes for MSM are not widely available despite evidence that such programmes may reduce HIV transmission.^9^

Oral antiretroviral pre-exposure prophylaxis (PrEP) is one of the most efficacious biomedical strategies in our toolkit for HIV prevention. Pre-exposure prophylaxis holds enormous potential to substantially reduce HIV acquisition in key populations globally.^9,10^ Among MSM, oral PrEP has been found to be particularly effective given sufficient drug concentrations, with prior research estimating a preventive efficacy of 96% – 100% with adherence to at least four doses of PrEP per week.^11,12^ Effective biomedical HIV prevention strategies, including PrEP, should be combined with evidence-based behavioural interventions to reduce HIV incidence on a population level.^13,14,15^ For PrEP rollout, however, research is needed to develop delivery programmes tailored to the unique needs of specific populations.^16^

The purpose of the Sibanye Health Project was to develop and evaluate a combination package of biomedical, behavioural, and community-level HIV prevention interventions, which included oral PrEP, for MSM and transgender women (TGW) in Cape Town and Port Elizabeth, South Africa.^17^ We examined PrEP uptake, adherence, and adverse events among HIV-negative South African MSM and TGW enrolled into the Sibanye cohort to inform intervention delivery, acceptability, and feasibility.

## Methods

### Screening and enrolment

The Sibanye Health Project (NCT02043015) was a prospective one-year pilot study evaluating the acceptability and uptake of a comprehensive combination package of HIV prevention services for MSM and TGW in South Africa. Services included condoms with condom-compatible lubricant choices, HIV testing with risk reduction counselling, couples HIV testing and counselling, screening for sexually transmitted infections (STIs), daily oral PrEP for interested and eligible participants, and non-occupational post-exposure prophylaxis. Methods for developing components of the prevention package have been described elsewhere.^17^

The Sibanye study recruited MSM and TGW living with and without HIV in Cape Town and Port Elizabeth from February 2015 to September 2015 using a combination of event- and venue-based, online, and participant referral recruitment methods. The study also recruited walk-in participants at the study clinics. Eligibility criteria included: age ≥ 18 years, self-reported anal intercourse with a man in the past year, current resident and planning to remain a resident for at least one year, literacy in English, Xhosa, or Afrikaans, male gender at birth, willing to provide contact information, and had a phone to facilitate scheduling visits.

Participants who tested HIV-negative at baseline were offered a combination HIV prevention package that included daily oral PrEP for those who were behaviourally and clinically eligible. Consistent with the 2012 South African HIV Clinician Society guidelines for safe use of oral PrEP in MSM at risk of HIV infection,^18^ to be behaviourally eligible for PrEP, participants had to meet one or more of the following HIV risk criteria as assessed by a provider: multiple partners, transactional sex, illicit drug use or abuse, heavy alcohol intake, at least one STI in the last year (including those diagnosed at screening), HIV-discordant relationship, and inability to use condoms consistently. Clinical eligibility criteria for PrEP included: no contraindications to tenofovir disoproxil fumarate/emtricitabine (TDF/FTC), creatinine clearance of ≥ 60 mL/min, aspartate aminotransferase/alanine aminotransferase (AST/ALT) < 2 × upper limit normal, hepatitis B surface antigen (HBsAg) negative, proteinuria and glycosuria < 2+, no history of diabetes diagnosis, and no signs of acute HIV infection or liver disease. Participants also had to report their willingness to attend visits at least every three months to be eligible for PrEP.

The Sibanye study enrolled participants prior to the approval of TDF/FTC for use as PrEP in South Africa. In December 2015, TDF/FTC was approved for use as PrEP by the South African Medicines Control Council (MCC) in combination with safer sexual practices, and in 2016, updated guidelines recommending PrEP for HIV-negative MSM in South Africa were published.^19^

### Pre-exposure prophylaxis delivery and monitoring

Different clinical models for PrEP delivery were used in the two cities.^17^ In Cape Town, PrEP was delivered through a gay-friendly research clinic located in an academic hospital that was previously providing HIV-related services to MSM. In Port Elizabeth, PrEP was delivered in three local public clinics; in each clinic, a designated room was used to provide all Sibanye services (including PrEP services) to participants, and all providers and clinic staff were trained in providing culturally competent care for MSM.

All Sibanye participants (regardless of PrEP uptake) attended a baseline visit and follow-up visits occurring at months 3, 6, and 12. HIV-negative participants who were interested in PrEP could be screened for PrEP eligibility at baseline or their month 3 visit. Eligible participants returned one month after screening (month 1 or month 4) to initiate PrEP. In addition to regular study follow-up visits, participants on PrEP had additional monitoring visits one month after initiating PrEP (month 2 or month 5) and at month 9.

At each visit following PrEP initiation, participants were monitored for side effects and had lab testing for HIV and to assess their creatinine level, liver enzymes (AST/ALT), phosphorus, proteinuria, and glycosuria. Adherence based on pill counts was calculated at each visit, and participants were provided with adherence counselling based on this data. Medication was dispensed to participants at all visits except the final visit. Providers could choose to see PrEP users more frequently for additional safety or adherence monitoring or to provide medication refills. Participants could also ‘drop in’ or schedule non-routine visits as needed during follow-up. In the Port Elizabeth clinics, providers often had participants come monthly throughout the study follow-up period for medication refills.

Participants could decide to stop PrEP at any time, and study providers could temporarily stop or permanently discontinue participants for clinical reasons. If not permanently discontinued, participants could restart PrEP provided they were still eligible and at the provider’s discretion. Participants who tested positive for HIV during the follow-up period were immediately discontinued from TDF/FTC, tested for drug resistance, and enrolled in care services. Dates of known PrEP stops and restarts were recorded on participant case report forms (CRFs). At the end of the study, participants on PrEP in Cape Town were referred to a non-governmental organisation serving MSM for PrEP continuation. At the time, Port Elizabeth did not have any local providers prescribing TDF/FTC for PrEP.

### Outcomes

Persistence on PrEP was assessed using dates of stops and restarts from participant CRFs. A PrEP stop was defined as the last date that PrEP was taken before ≥ 14 days off of PrEP following initiation, consistent with previous research and when protection with PrEP is estimated to be low.^20,21^ Permanent stops, or PrEP discontinuation, was defined as the last date PrEP was taken without restarting before study end. For participants with an uncertain PrEP discontinuation date, the halfway point between their last known date on PrEP and the date they were known to not be on PrEP was used. Self-reported reasons for permanent stops were collected by study staff on a CRF at the time of discontinuation. A participant was categorised as persistent to study end if they picked up PrEP at their month 9 visit or restarted PrEP following their month 9 visit prior to study end.

Adherence was measured through the self-reported number of pills missed in the last seven days and by a pill count, measured as pills dispensed minus pills returned, divided by the number of days since the dispense date, and was assessed at all follow-up visits while a participant was taking PrEP. Thresholds for high adherence included ≥ 4 doses of PrEP self-reported to be taken per week and ≥ 57% of pills taken per week as determined by pill counts. The thresholds were associated with 100% protection against HIV acquisition among MSM in the iPrEX OLE study.^11,12^ For Cape Town participants only, dried blood spots (DBS) collected at each subsequent visit after PrEP initiation were used to test intracellular concentrations of tenofovir-diphosphate (TFV-DP). Fifty microliters of whole blood was spotted on filter paper, dried at room temperature for 2 hours, sealed in bags with desiccant, and stored at −80 °C until analysis. An indirect method for the quantification of TFV-DP in 50 µL DBS was developed and validated at the Division of Clinical Pharmacology, University of Cape Town.^22^ Dried blood spots provide a measure of cumulative adherence in the month prior to sample collection.^23^ Thresholds for high (≥ 700 fmol/punch; ≥ 4 pills taken per week), medium (350 fmol/punch – 699 fmol/punch; 2–3 pills taken per week), and low (< 350 fmol/punch; < 2 pills taken per week) adherence^24^ were used to compare TFV-DP concentrations to self-report and pill-count measures.

Adverse events were assessed at routine and non-routine visits and were recorded on participant CRFs by grade (mild, moderate, severe) and potential relation to PrEP. Adverse events were specified by participant self-report or by a provider based on clinical or lab assessments. Lastly, patterns of PrEP use and discontinuation among participants who seroconverted during the study were described.

### Analysis

The authors described the continuum of HIV-negative participants enrolled into the Sibanye cohort from diagnosis to PrEP initiation. A descriptive summary of socio-demographic and behavioural characteristics was conducted for HIV-negative participants, participants who initiated PrEP, and participants who did not initiate PrEP at baseline. Participants who did and did not initiate PrEP were compared across characteristics using a Fisher’s exact test. To determine the proportion of available days that participants were on and off PrEP, the median number of days on PrEP (excluding stops) and median number of available PrEP days during follow-up were calculated. Kaplan-Meier estimates were used to describe the time from initiation to first PrEP stop and PrEP discontinuation (last stop). Participants who did not stop PrEP early were censored at study end even if they had continued PrEP as part of their medical care. Patterns of PrEP stops and restarts were described for all participants who initiated PrEP. Adherence to PrEP was described using multiple measures (self-report, pill-count, TFV-DP) at routine monitoring visits only. Assessments during known PrEP stops and after discontinuation were excluded. Kappa statistics were used to assess the agreement between self-report and pill count adherence measures.^24^

### Ethical considerations

This study was approved by the Institutional Review Board of Emory University (protocol IRB00054229), the Human Research Ethics Committee of the University of Cape Town, the Research Ethics Committee of the Human Sciences Research Council, and the South African MCC. All participants enrolled into the Sibanye Health Project provided written informed consent prior to participation.

## Results

**FIGURE 1:**
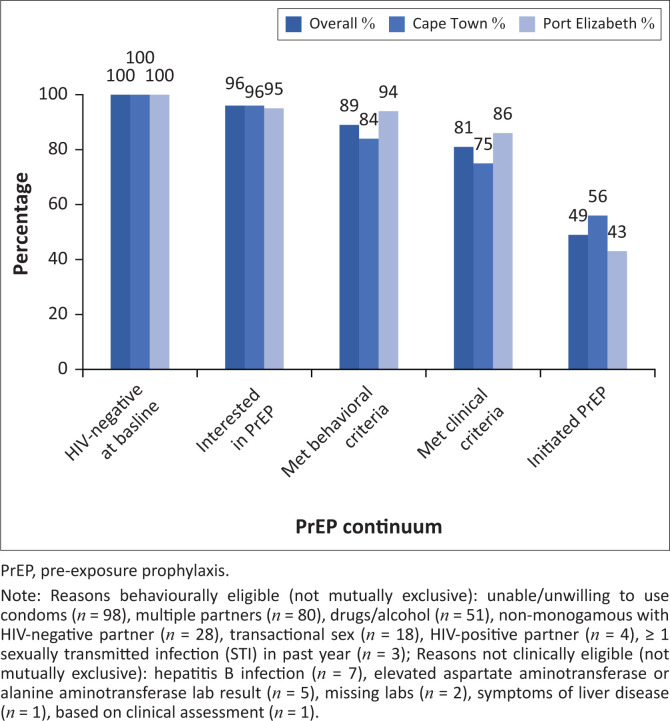
Pre-exposure prophylaxis continuum among South African men who have sex with men and transgender women in a combination HIV prevention trial, 2015.

Among participants screened for the study, 167 participants tested HIV-negative at baseline (80 from Cape Town and 87 from Port Elizabeth), of whom 160 (96%) were interested in PrEP and evaluated for PrEP eligibility. Among these, 135 were behaviourally and clinically eligible (84% eligibility). Between month one and month four, 82 participants initiated PrEP (61% uptake), including 45/60 participants from Cape Town (75%) and 37/75 participants from Port Elizabeth (49%). Four participants who were interested in PrEP and who screened as behaviourally and clinically eligible at baseline did not return for their one-month visit. A continuum of participant drop-off from testing to uptake is shown in Figure 1. Participants who initiated PrEP were primarily Black African, under 30 years of age, and male-identifying. There were no significant socio-demographic and behavioural differences between participants who initiated PrEP and those who did not (Table 1).

**TABLE 1:** Baseline socio-demographic and behavioural characteristics among South African men who have sex with men and transgender women in a combination HIV prevention trial, 2015.

Characteristic	HIV-negative		Ever initiated PrEP		Did not initiate PrEP†		Initiated versus not – *P-value*
	*n*	%	*n*	%	*n*	%	
**Overall**	167	100	82	49	85	51	-
**Site**							0.089
Cape Town	80	48	45	55	35	41	-
Port Elizabeth	87	52	37	45	50	59	-
**Age range**							0.137
18–19	28	17	13	16	15	18	-
20–24	75	45	35	43	40	47	-
25–29	29	17	11	13	18	21	-
30+	35	21	23	28	12	14	-
**Race**							0.596
Black person	137	82	65	79	72	85	-
Mixed race person	27	16	15	18	12	14	-
Other	3	2	2	2	1	1	-
**Gender identity**							1.000
Male	153	92	77	94	76	89	-
Other (female, transgender)	9	5	4	5	5	6	-
**Sexual orientation**							0.713
Homosexual or gay	87	52	45	55	42	49	-
Bisexual	61	37	29	35	32	38	-
Straight or other	15	9	6	7	9	11	-
**Education**							0.264
Primary incomplete	83	50	35	43	48	56	-
Primary complete	82	49	45	55	37	44	-
**Work/student status**							0.876
Part/full-time student or job	84	50	42	51	42	49	-
Not a student and no job	79	47	38	46	41	48	-
**Income**							0.510
No income	91	54	41	50	50	59	-
R1.00 – R4800.00	38	23	21	26	17	20	-
R4801.00 +	38	23	20	24	18	21	-
**Receptive condomless anal intercourse, past 3 months**							0.169
No	109	65	51	62	58	68	-
Yes	34	20	21	26	13	15	-
**Number of male partners, past 3 months**							0.920
0	13	8	6	7	7	8	-
1–2	104	62	52	63	52	61	-
3+	22	13	10	12	12	14	-
**Any female partners, past 12 months**							0.609
No	117	70	55	67	62	73	-
Yes	48	29	25	30	23	27	-
**Transactional sex, past 12 months**							0.404
No	124	74	62	76	62	73	-
Yes	27	16	11	13	16	19	-
**Injection drug use, past 6 months**							0.670
No	52	31	25	30	27	32	-
Yes	5	3	3	4	2	2	-
**Any drug use, past 6 months**							1.000
No	109	65	53	65	56	66	-
Yes	57	34	28	34	29	34	-
**Binge drinking (5+ drinks) on 5 or more days, past 30 days**							0.291
No	126	75	59	72	67	79	-
Yes	27	16	16	20	11	13	-

Note: *P*-values calculated using Fisher’s exact test.

PrEP, pre-exposure prophylaxis.

† , Among participants who did not initiate PrEP, 32 (20 Cape Town, 12 Port Elizabeth) were not eligible.

**FIGURE 2:**
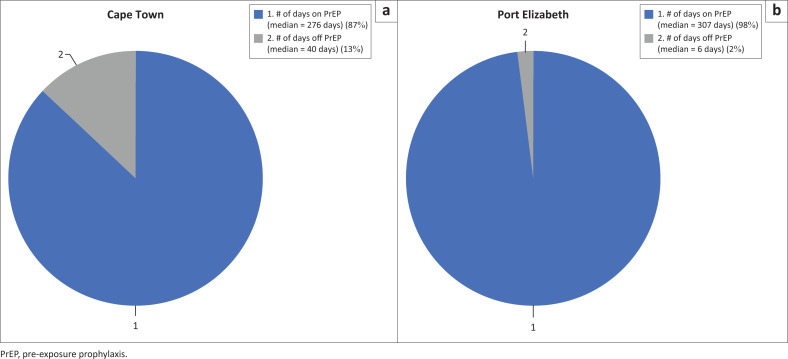
Proportion of available days on and off pre-exposure prophylaxis by study site among South African men who have sex with men and transgender women in a combination HIV prevention trial, 2015.

Most participants (65/82) initiated PrEP at their month 1 visit. Participants who initiated PrEP were on PrEP for a median of 294 days (interquartile range [IQR]: 217–319 days) out of a median of 314.5 possible days (IQR: 252–329 days) (Figure 2). Thus, participants were on PrEP for a total of approximately 93% of available PrEP days. The median number of visits for participants in the 11 months after PrEP initiation (including regular study visits and drop-in visits) was seven (IQR: 6–9). A total of 67 participants (82%) picked up PrEP at their month 9 visit or, if they were not on PrEP during their month 9 visit, had restarted PrEP prior to study end and were thus considered to be persistent to study end, including 34/45 participants from Cape Town (76%) and 33/37 participants from Port Elizabeth (89%). Early PrEP discontinuation (*n* = 15) was most commonly reported by participants to be the result of side effects (*n* = 9) and was initiated by both participants (*n* = 6) and clinicians (*n* = 3). Other self-reported reasons for PrEP discontinuation included family relationships (*n* = 2), missing medication pick-up (*n* = 1), no longer interested (*n* = 1), moving out of the study area (*n* = 1), and HIV seroconversion (*n* = 1).

**FIGURE 3:**
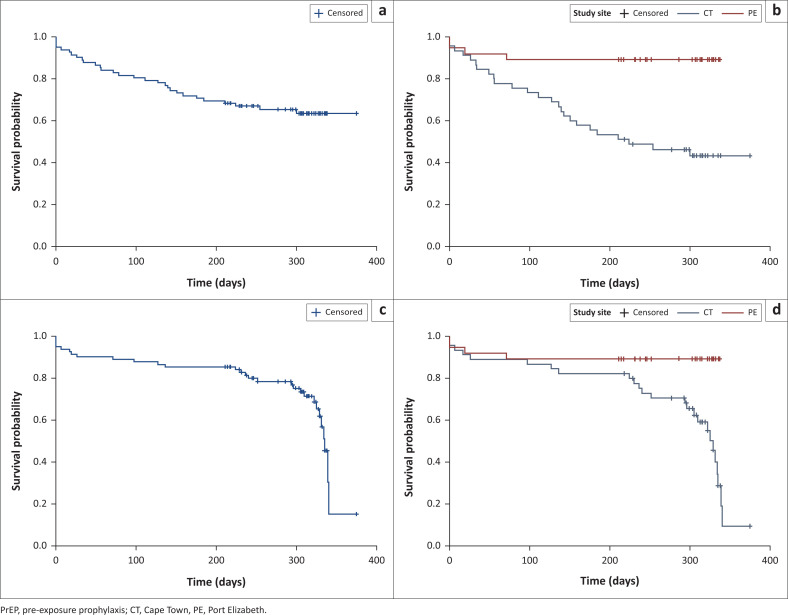
(a, b) Kaplan-Meier survival analysis for time to first pre-exposure prophylaxis stop or study end (for those who did not stop pre-exposure prophylaxis), overall and by site among South African men who have sex with men and transgender women in a combination HIV prevention trial, 2015. (c, d) Kaplan-Meier survival analysis for time to last pre-exposure prophylaxis stop or study end (for those who did not stop pre-exposure prophylaxis), overall and by site among South African men who have sex with men and transgender women in a combination HIV prevention trial, 2015.

**FIGURE 4:**
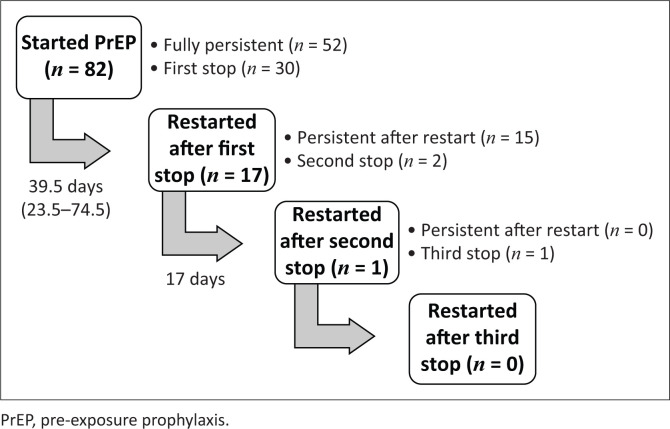
Patterns of pre-exposure prophylaxis stops and restarts for all pre-exposure prophylaxis initiators among South African men who have sex with men and transgender women in a combination HIV prevention trial, 2015. Each stop represents ≥ 14-days off pre-exposure prophylaxis. Time in days is median time from pre-exposure prophylaxis stop to restart and interquartile range.

Over one-third of participants (37%; 30/82) had at least one known PrEP stop. The median time from PrEP initiation to first stop (≥ 14 days) was 265.5 days (IQR: 142–316 days) in the full cohort, 218 days (IQR: 97–305 days) in Cape Town, and 307 days (IQR: 232–324 days) in Port Elizabeth (Figure 3a). Further, the median time from PrEP initiation to discontinuation (i.e. last stop) was 304.5 days (IQR: 232–325 days) in the full cohort, 304 days (IQR: 230–325 days) in Cape Town, and 307 days (IQR: 232–324 days) in Port Elizabeth (Figure 3b). Twenty-eight participants stopped PrEP only once before study end, of whom 13 stopped permanently (i.e. discontinued PrEP) and 15 restarted PrEP and were persistent to study end. Two additional participants stopped PrEP more than once. These participants permanently discontinued PrEP at their second and third stop (Figure 4).

**FIGURE 5:**
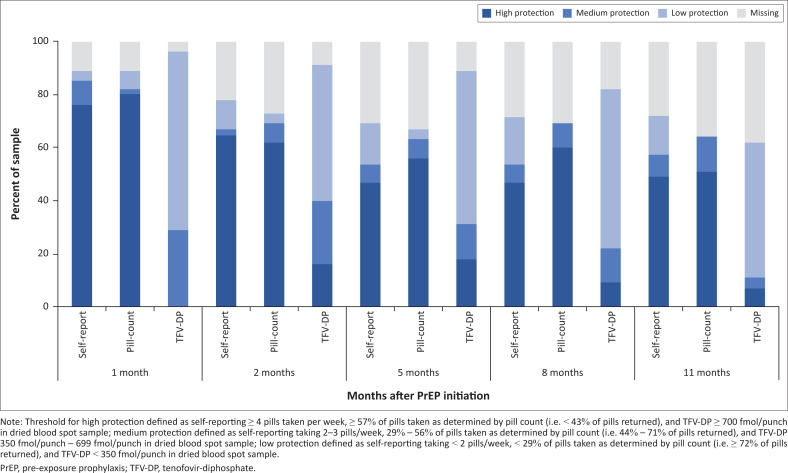
Oral HIV pre-exposure prophylaxis adherence measures among 45 men who have sex with men and transgender women from Cape Town, South Africa in a combination HIV prevention trial, 2015.

Across the follow-up time points (1–11 months post-PrEP-initiation), 57% – 72% of participants self-reported taking protective levels of PrEP, and 59% – 74% were adherent to PrEP as indicated by pill counts (Table 2). Results of quantitative TFV-DP concentrations by DBS testing (available for Cape Town only, online Appendix 1, Table 1-A1) indicated that no participants had protective concentrations of TFV-DP (≥ 700 fmol/punch) one month after initiating PrEP, 16% (7/45) had protective levels after two months, 18% (8/45) after 5 months, 9% (4/45) after eight months, and 7% (3/45) after 11 months (Figure 5). Additionally, 29% (13/45) of participants had TFV-DP levels from 350 fmol/punch to < 700 fmol/punch one month after initiating PrEP, 24% (11/45) after two months, 13% (6/45) after five and eight months, and 4% (2/45) after 11 months.

**TABLE 2:** Self-reported and pill-count adherence among South African men who have sex with men and transgender women in a combination HIV prevention trial, 2015.

Adherence measure	1 month after initiating PrEP				2 months after initiating PrEP				5 months after initiating PrEP				8 months after initiating PrEP				11 months after initiating PrEP			
	*n*	%	*k*	95% CI	*n*	%	*k*	95% CI	*n*	%	*k*	95% CI	*n*	%	*k*	95% CI	*n*	%	*k*	95% CI
**Self-reported adherence (*n* = 82)**																				
≥ 4 pills/week	59	72	-	-	54	66	-	-	47	57	-	-	49	60	-	-	39	60	-	-
< 4 pills/week	9	11	-	-	11	13	-	-	15	18	-	-	15	18	-	-	12	18	-	-
Missing	14	17	-	-	17	21	-	-	20	24	-	-	18	22	-	-	14	22	-	-
**Pill-count adherence (*n* = 82)**																				
≥ 57% of pills taken	61	74	-	-	48	59	-	-	48	59	-	-	56	68	-	-	40	62	-	-
< 57% of pills taken	9	11	-	-	15	18	-	-	12	15	-	-	7	9	-	-	8	12	-	-
Missing	12	15	-	-	19	23	-	-	22	27	-	-	19	23	-	-	17	26	-	-
**Kappa statistic comparing reliability of self-report to pill-count adherence**	-	-	0.39	0.07, 0.72	-	-	0.36	0.08, 0.63	-	-	0.51	0.25, 0.77	-	-	0.53	0.26, 0.81	-	-	0.45	0.14, 0.77

Note: Denominator is total PrEP initiators (*n* = 82) at each time point except for 11 months after initiation, since only month-1 initiators were on PrEP for 11 months (*n* = 65); adherence measures after a known permanent PrEP stop or during a temporary PrEP stop were excluded; missing data may also be because of a missed visit or a missing case report form (CRF).

PrEP, pre-exposure prophylaxis.

A total of 41 side effects were reported during the study among 28 participants (online Appendix 1, Table 2-A1). Side effects that were most commonly reported included nausea (*n* = 6), fatigue (*n* = 6), proteinuria (*n* = 5), glycosuria (*n* = 4), heartburn (*n* = 4), and increased thirst or dry mouth (*n* = 4). The majority of the side effects were judged to be mild (*n* = 29) or moderate (*n* = 9) by study clinicians. Two participants had elevated ALT/AST levels, which were rated as severe. One side effect (nausea) was missing a severity rating. Pre-exposure prophylaxis was held by study clinicians due to side effects, either permanently or temporarily, for 18 participants (22%) during the study.

Among participants who initiated PrEP, there were five HIV seroconversions (6%) during study follow-up, all in Cape Town (online Appendix 1, Table 3-A1). Among these, two participants initiated permanent PrEP stops ≥ 30 days before their HIV-positive test results due to side effects. The first stopped PrEP six days following initiation due to a mild hypersensitivity reaction and was diagnosed with HIV 55 days later. The second participant stopped PrEP the same day they initiated due to gastrointestinal (GI) problems and was diagnosed after approximately one year. One additional participant stopped PrEP approximately four months following initiation due to GI problems and was diagnosed with HIV 23 days later. Lastly, two participants had not stopped PrEP before their HIV diagnoses and were diagnosed 316 days and 303 days following PrEP initiation. Both participants had low adherence at their diagnosis visit, as indicated by TFV-DP concentrations at their month 12 visit (130 fmol/punch and 0 fmol/punch).

## Discussion

The Sibanye Health Project was a prospective one-year pilot study evaluating the acceptability and uptake of a comprehensive HIV prevention package that included daily oral PrEP for MSM and TGW in South Africa. Approximately half of the participants who tested HIV-negative at baseline initiated PrEP during the study despite approximately 80% being behaviourally and clinically eligible and interested in PrEP. Uptake was lower than observed among Kenyan MSM, where over 80% of participants initiated PrEP.^25^ In the Kenyan study, eligible MSM who were willing to start PrEP could initiate PrEP right away, while MSM in the Sibanye Health Project had to wait one month to initiate PrEP following confirmation of their lab results. Pre-exposure prophylaxis should be available to MSM to initiate immediately following an HIV-negative diagnosis to avoid potential fallout between initial screening and PrEP uptake.^26^

Among those who initiated PrEP, over 80% were persistent with PrEP at study end. This estimate was higher than in Kenya, where only 46% of MSM who initiated PrEP reported use at study end.^25^ Adherence in the Cape Town cohort, however, was relatively poor by objective laboratory measures, with 18% of participants or fewer having achieved protective TFV-DP concentrations at any time point. Self-report and pill count adherence assessments were higher (57% – 74%) across time points. Overestimation of adherence due to recall or social desirability bias has been observed in prior PrEP studies.^27,28,29,30^ However, it is possible that there were unknown factors related to DBS collection or storage that negatively impacted the quality of the study samples and, thus, our findings. Future research should compare self-report and pill count adherence measures with objective biological measures among South African MSM and TGW to provide additional insight into the conflicting estimates observed across measures in this study. Given the high level of PrEP interest and likely challenges with daily adherence, the acceptability of longer-acting PrEP formulations should be examined.

Although side effects were typically mild and often resolved for men who remained on PrEP, for 18 participants (22%), PrEP was held by study clinicians, either permanently or temporarily, when the side effect was reported. Side effects were the most common reason for early PrEP discontinuation cited by participants at their exit visit. Among the five PrEP initiators who seroconverted during the study, three had previously discontinued PrEP due to side effects (of whom two were due to GI side effects). Thus, adherence counselling for dealing with side effects, and especially GI upset, is likely important for MSM taking PrEP. Further, providers prescribing PrEP should consider if the risks associated with certain side effects outweigh the risks associated with stopping PrEP, even temporarily.

Different models of delivery were used in Cape Town and Port Elizabeth. While PrEP uptake was higher in Cape Town, participants in Port Elizabeth were on PrEP for a greater number of days, and fewer participants discontinued PrEP before study end. The successful experience of implementing a PrEP programme for MSM and TGW in district health clinics in Port Elizabeth provides evidence that specialised facilities are not required to provide confidential, acceptable, and high-quality PrEP services, but that provision of PrEP in public health clinics is likely a feasible approach to reach those at highest risk for HIV infection.

The Sibanye Health Project began enrolling MSM and TGW in 2015 prior to the approval of TDF/FTC as PrEP by the South African MCC. Despite PrEP’s novelty in South Africa, many MSM and TGW were interested in PrEP; over half of MSM and TGW initiated PrEP, and the majority persisted with PrEP to study end. Notably, these early efforts to provide PrEP to HIV-negative MSM and TGW resulted in higher uptake and persistence than similar programmes reported in the United States^21,31^ and Europe.^32^ Since 2015, PrEP has gained recognition in South Africa as a safe and effective HIV prevention strategy with utility across key populations. South African MSM, however, still face high levels of stigma and discrimination that make it difficult to access healthcare services.^8^ Thus, research is still needed to explore methods for reaching South African MSM and TGW who may benefit from PrEP and to develop tailored adherence programmes.

Our study has limitations. Due to this study’s sampling methods, findings from this study may not be generalisable to all South African MSM and TGW. Although this study describes patterns of PrEP use behaviour, the main goal of the Sibanye Health Project was not to track PrEP stops and restarts, and thus this data may have been imprecisely captured. Participants who initiated PrEP were not rescreened for HIV risk during follow-up. Risk can be dynamic, particularly in young populations, and it could be that poor adherence or discontinuation of PrEP was the result of reductions in perceived levels of HIV risk. PrEP estimates should be interpreted in the context of the other services provided in the Sibanye Health Project; PrEP uptake and persistence, for example, might be less in the absence of supportive services, including opportunities for drop-in visits, free access to other prevention commodities, risk reduction counselling, and the availability of providers trained in cultural competency and PrEP provision. Finally, data collection began in 2015, before the regulatory approval of PrEP in South Africa. Pre-exposure prophylaxis delivery to MSM and TGW in Africa remains sparse, and research exploring the acceptability and feasibility of oral PrEP for MSM and TGW in South Africa is still needed.

## Conclusion

Our findings suggest that PrEP can be successfully delivered to MSM and TGW in South Africa in diverse programmatic contexts. The MSM and TGW in the Sibanye Health Project showed a high interest in PrEP and high persistence on PrEP over one year. Uptake of PrEP, however, was low, and discrepancies between self-report and biological adherence measures suggest there are adherence challenges for MSM and TGW taking PrEP. Counselling for coping with side effects and motivating daily pill taking is recommended to support South African MSM and TGW in achieving protection with PrEP. Research is needed on interventions to improve PrEP uptake and adherence and to examine the acceptability of alternative PrEP formulations.
